# Supplementary material for: Physics-based forecasting of man-made earthquake hazards in Oklahoma and Kansas
Source: Nat Commun. 2018 Sep 26;9:3946. doi: 10.1038/s41467-018-06167-4 (PMC6158231; doi:10.1038/s41467-018-06167-4)
Supplement: Supplementary file 1 — Supplementary Information [file 41467_2018_6167_MOESM1_ESM.pdf]

Supplementary Information

Physics-based forecasting of man-made earthquake hazards in Oklahoma and Kansas

Langenbruch et al.

Modeled Unit	Porosity	Fluid Density (kg m^{-3})	Fluid Viscosity (cP)	B_w (Pa^{-1})*	B_r (Pa^{-1})**	S_s (m^{-1})***
**Arbuckle Group**	0.2[a]	1062[c]	0.547[d]	4.4×10^{-10}	0.16×10^{-10}	1×10^{-6}
**Crystalline Basement**	0.01[b]	1062[c]	0.547[d]	4.4×10^{-10}	0.7×10^{-11}	1×10^{-7}

[a]From range of reported values[1,2]
[b]From range of reported values[3]
[c]Calculated assuming a reservoir temperature of 50 °C and brine TDS of 100,000 ppm [4]
[d]Calculated assuming a reservoir temperature of 50 °C and brine TDS of 100,000 ppm [4]
*Bulk compressibility of water assuming a reservoir temperature of 50 °C
**Bulk compressibility of Arbuckle Group[5] and crystalline basement[3]
***Specific Storage for all model sensitivity analysis

Supplementary Table 1. Hydrogeologic model material property inputs. The porosity, fluid density, viscosity, fluid compressibility, rock compressibility and specific storage used for each unit in the model.

Model Run	Arbuckle Group (2.1 – 2.5 km depth)	Crystalline Basement (2.5 – 20 km depth)	Nemaha Fault Zone (2.1 – 20 km depth)
1	10^{-12}	2×10^{-14}	10^{-20}
2	10^{-12}	10^{-14}	10^{-20}
Preferred Model	10^{-12}	2×10^{-15}	10^{-20}
4	10^{-12}	1.5×10^{-15}	10^{-20}
5	10^{-12}	10^{-15}	10^{-20}
6	10^{-12}	10^{-16}	10^{-20}
7	10^{-13}	2×10^{-15}	10^{-20}
8	10^{-14}	2×10^{-15}	10^{-20}

Supplementary Table 2. Model sensitivity analysis of hydrogeologic permeability. Parameterization of the layered permeability heterogeneity of eight hydrogeologic model runs. Permeability is measured in m^2**.**

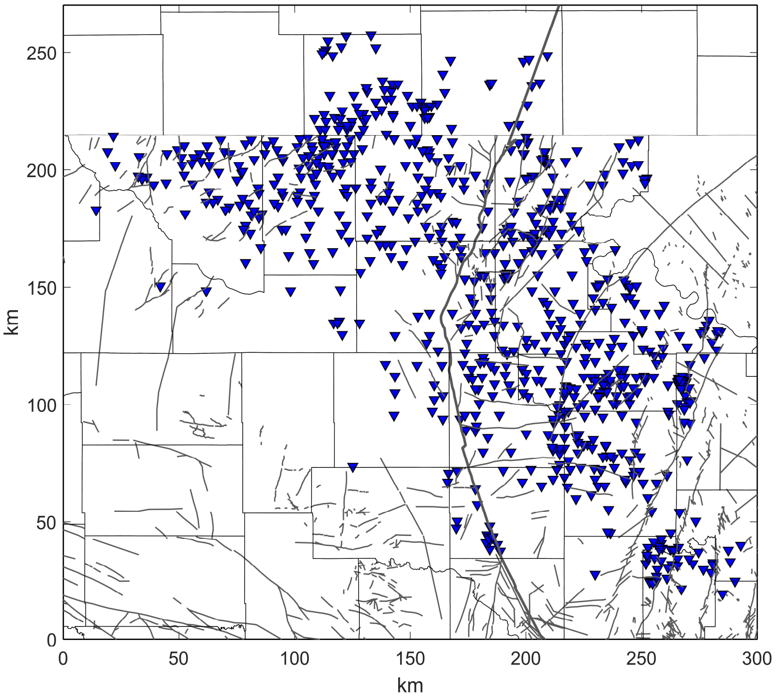

Supplementary Figure 1. Locations of Arbuckle saltwater disposal wells considered in the study.

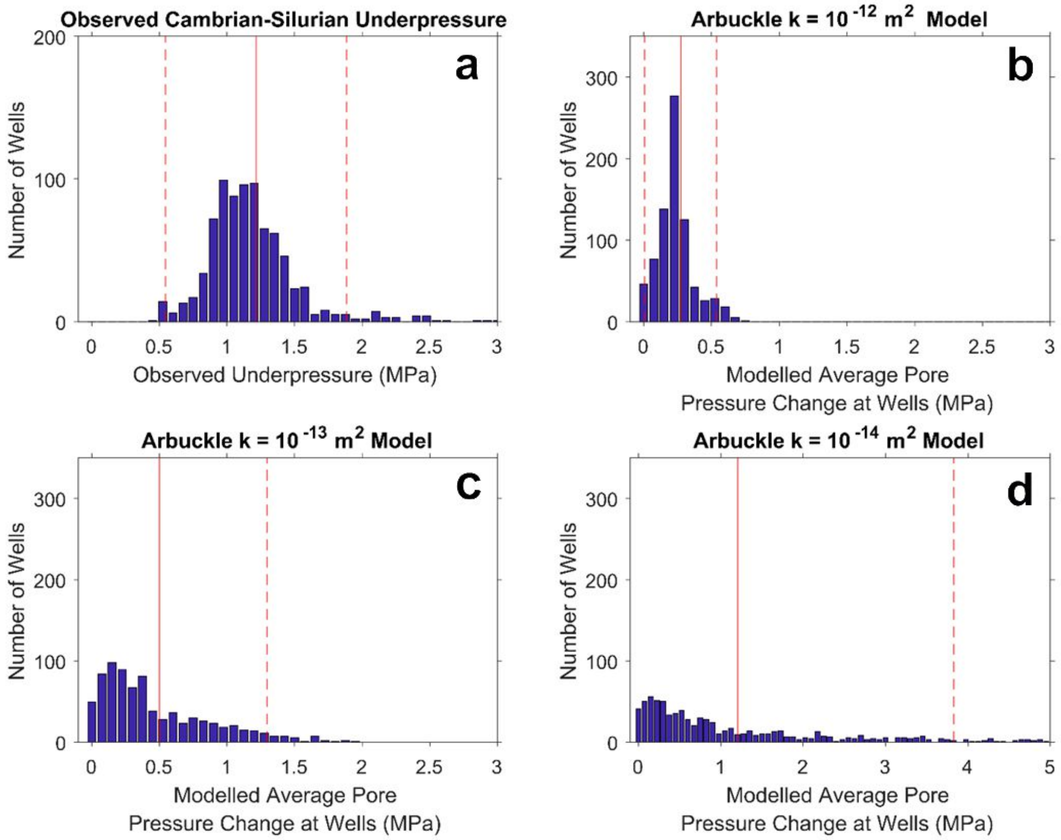

Supplementary Figure 2. Observed underpressure and modelled average pore pressure changes at injection wells. Histograms of (**a**) observed hydraulic underpressure in the Area of Interest region of Oklahoma using data derived from Nelson et al. (2015)[42]. Hydraulic underpressure is the difference between the ambient fluid pressure in formations of Cambrian-Ordivician-Silurian age and the land surface elevation. Mean underpressure across the Area of Interest is 1.2 MPa (solid red line) and varies spatially, but is generally between 0.5 and 2 MPa. (**b**) Modelled average pore pressure change at Arbuckle injection wells in 2015-2016 using an Arbuckle permeability of 10^{-12} m^2. (**c**) Modelled average pore pressure change at Arbuckle injection wells in 2015-2016 using an Arbuckle permeability of 10^{-14} m^2. (**d**) Modelled average pore pressure change at Arbuckle injection wells in 2015-2016 using an Arbuckle permeability of 10^{-14} m^2. Solid red lines show the mean of the distribution. Dashed red lines show 2 standard deviations from the mean.

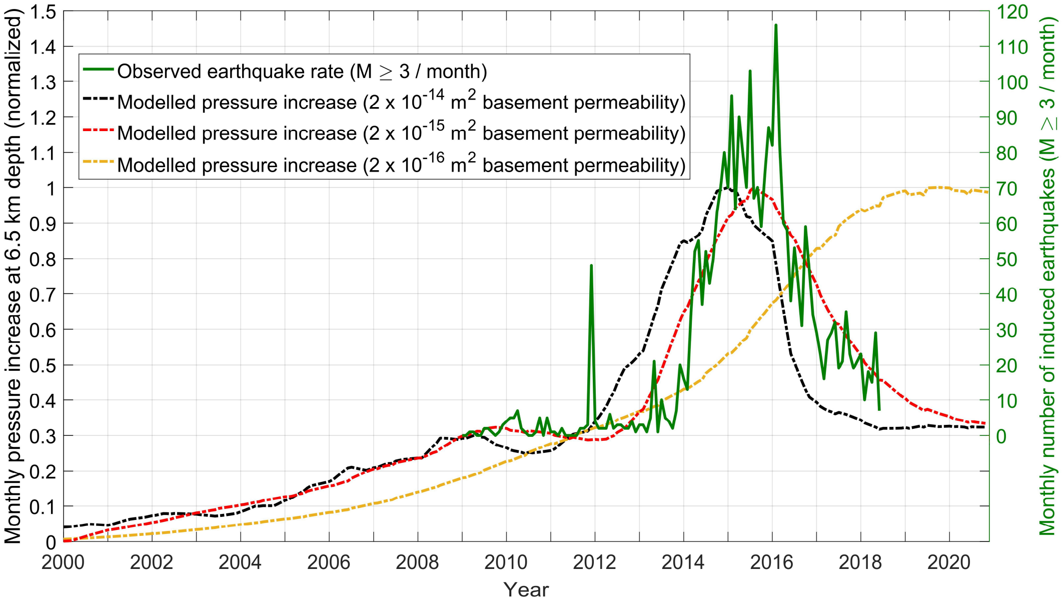

Supplementary Figure 3. Seismicity-based calibration of basement permeability in Oklahoma and Kansas. Based on the concept that changes of the earthquake rate (green) are caused by changes of the rate of pressure increase (dashed coloured lines), we vary the basement permeability in our model and compare average monthly pressure increase at all 25000 seed points to the overall observed monthly earthquake rate ($M \geq 3$). Monthly pressure increase is normalized to the maximum of modelled monthly rates. The pressure increase at seismogenic depth (dashed lines) mimics the shape of the injection rate (see Fig. 1b), but there is a delay time controlled by basement permeability. A basement permeability of 2 x 10^{-15} m^2 best fits the shape of the earthquake rate and is considered in our hydrogeologic model.

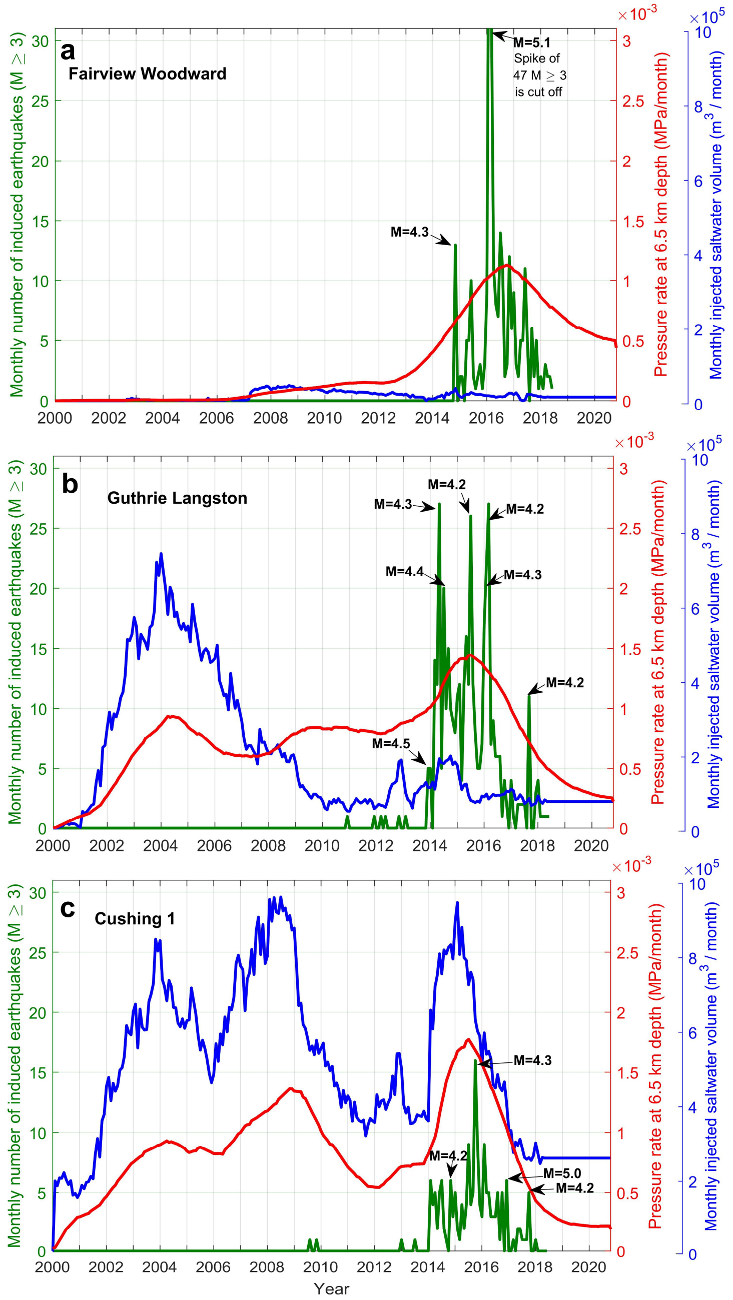

Supplementary Figure 4. Reported injection rates, modelled pressure increase and observed seismicity rates in three local-scale study regions. Intensity and temporal evolution of earthquake rates are unrelated to local-scale injection rates. Time-lags of up to ten years between peak injection and peak seismicity rates are observed. While local scale injection and seismicity is unrelated, observed peak seismicity rates occur at peak pressure rates resulting from our model. Far-field pressurization, caused by high rate injection wells outside of the considered areas, dominates and explains the high time lags between local injection and seismicity. An open system hydrogeologic model and high Arbuckle permeability are essential to forecast local-scale seismicity rates. The locations of the three regions are shown in Supplementary Fig. 6.

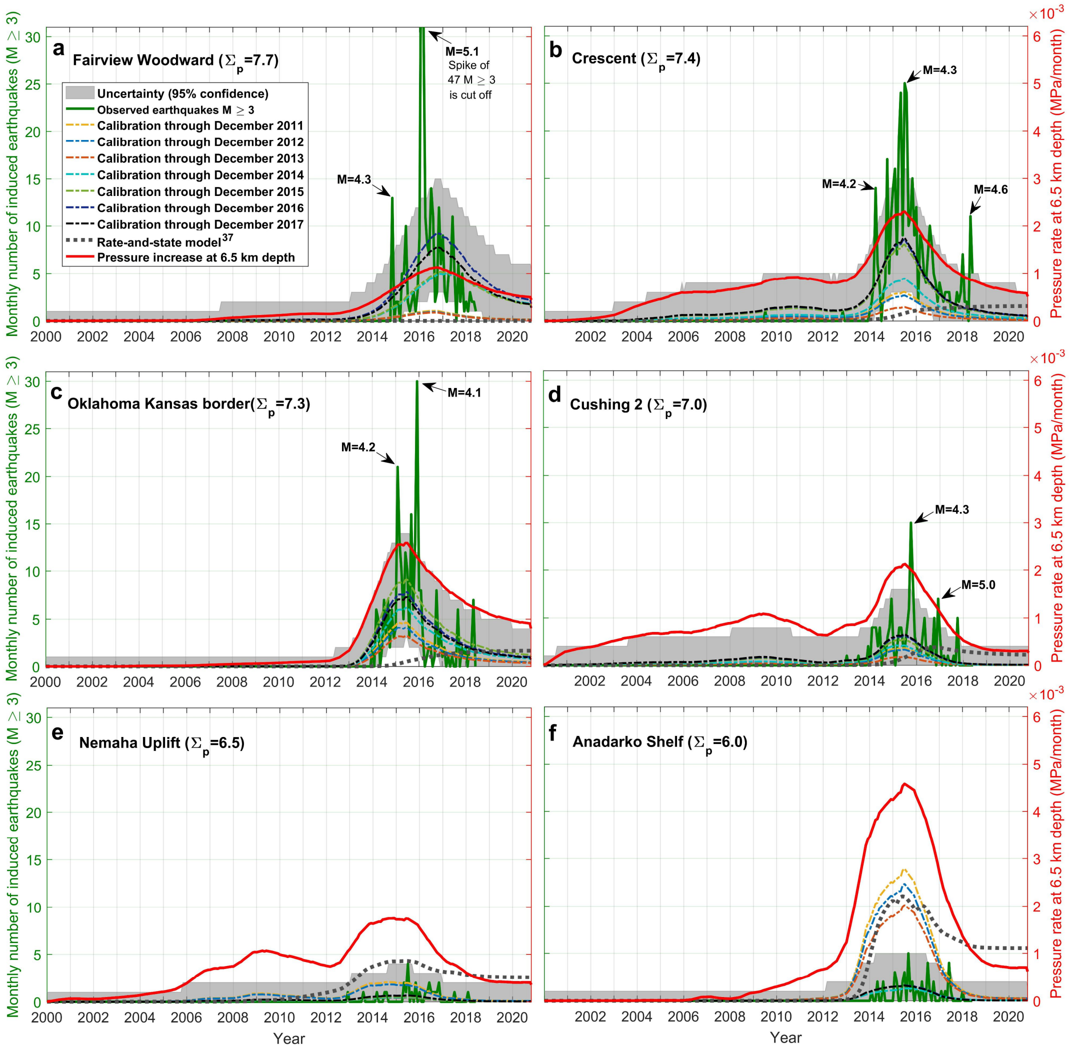

Supplementary Figure 5. Performance evaluation of the SI model in six local-scale regions of 25 km radius. a-f: Observed earthquakes rates, modelled pressure rates and calibrated SI models. Panels **a-f** are sorted in descending order of the SI in the local-scale regions. Observed peak seismicity rates (green) in all areas occur at modelled peak pressure rates (red). However, because the regions are characterized by different SI ranging from 6.0 (Anadarko Shelf) to 7.7 (Fairview Woodward) the same pressure rate is causing a significantly different number of earthquakes. In the Fairview Woodward region almost a hundred times more earthquakes are caused by the same pressure increase compared to the Anadarko Shelf region. Accurate prediction of local scale seismicity requires model calibration on the local scale. As soon as seismicity occurs in a local-scale region the local-scale SI can be computed and the forecast of the model becomes accurate and stable. Spikes caused by aftershocks peak out of the 95% confidence interval of the model forecasts. We also present a comparison to a recently published rate and state model[37]. Because this model does not take into account spatially variable model parameter it is unable to predict onset, increase, peak or decrease of seismicity rates in the local-scale regions.

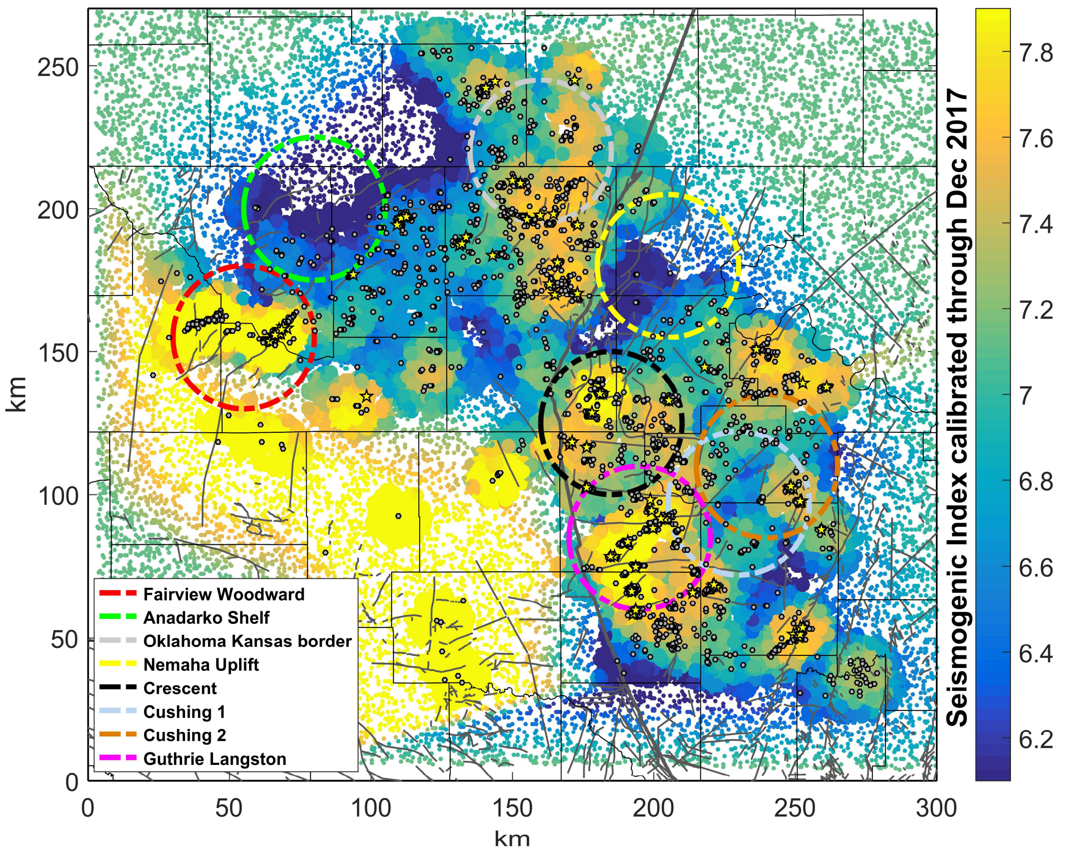

Supplementary Figure 6. | Seismogenic Index map showing the local-scale study regions analysed in Supplementary Fig. 4 and 5. The local-scale regions are chosen to represent the full value range of the SI observed in Oklahoma and Kansas. Grey circles and yellow stars show M ≥ 3 and M ≥ 4 earthquakes, respectively. In dotted areas no sufficient seismicity data exists to directly calibrate the SI based on observed earthquakes and modelled pressure rates. We apply the following rule to determine SI values in these regions. If no directly calibrated SI value exist within 40 km radius around a selected seed point, the SI is set to the mean value of all directly calibrated SI values. If directly calibrated SI values exist within 40 km radius, the SI is interpolated.

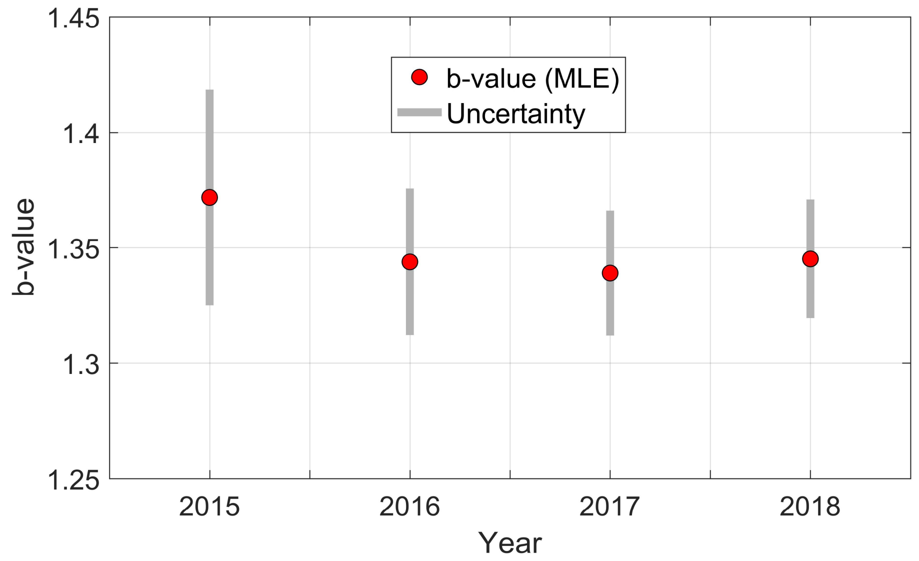

Supplementary Figure 7. Maximum likelihood estimate of the b-value. The b-values have been computed according to the complete earthquake catalogue at time given by the x-axis. Fluctuations of the b-value occur within the uncertainty of the computation.

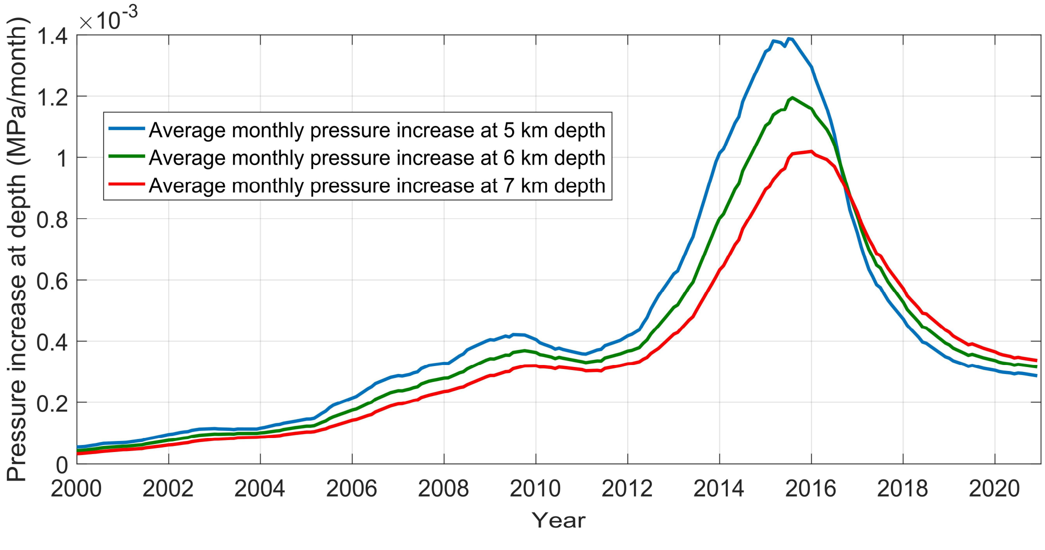

Supplementary Figure 8. Modelled rate of pressure increase at 5, 6 and 7 km depth. In response to decreased saltwater injection rates, pressure increases are slowing down over a wide range of depth.

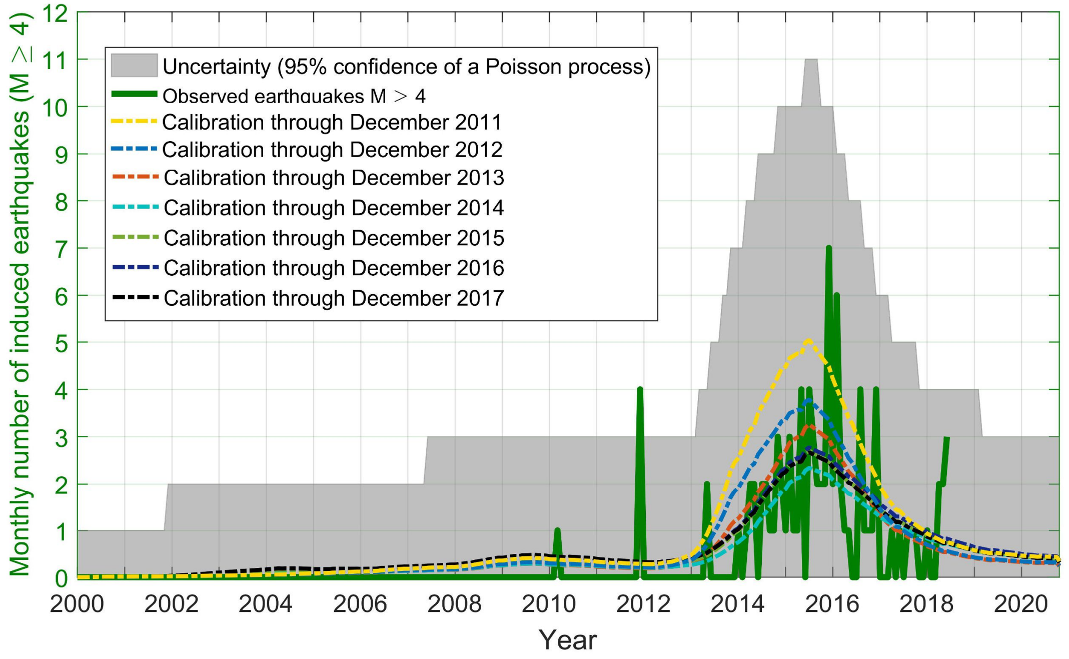

Supplementary Figure 9. Forecasted seismicity rate (M ≥ 4) in the complete study area. Observed earthquake rate (solid green line), forecasted seismicity rates resulting from SI models calibrated through different temporal endpoints (Dec 2011 - Dec 2017) (coloured dashed lines). While the observed rate of M ≥ 4 earthquakes falls well within the uncertainty range of our model, it does not allow us to draw statistically significant conclusions, because of the large uncertainty related to the small number of M ≥ 4 observations.

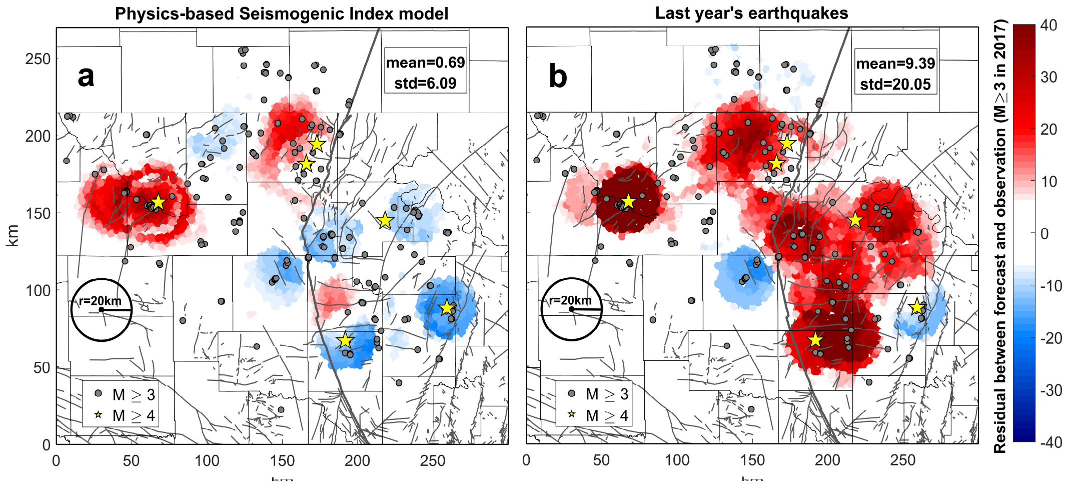

Supplementary Figure 10. Performance evaluation of the 2017 forecast in space. Residuals between 2017 forecast and observation of M ≥ 3 in areas of 20 km radius are shown for **a** our physics-based Seismogenic Index model and **b** the observational approach of using last year's earthquakes to predict next years's seismicity. Over-prediction (red), under-prediction (blue). Our physics-based approach (**a**) significantly reduces the residuals between forecast and observation. The mean residual value close to zero (mean = 0.69 M ≥ 3 earthquakes) points out that, on average, our model is correctly forecasting the number of earthquakes in space. Changes of earthquake clustering are captured in our model by introducing a time and space dependent earthquake productivity. In contrast, using last year's earthquakes to predict next year's earthquakes, systematically over-predicts the number of earthquakes in 2017 (mean = 9.39 M ≥ 3 earthquakes). Moreover, the standard deviation of the residuals is significantly decreased from 20.05 to 6.09 by incorporating injection rates through our physics-based approach. It also becomes apparent that, as predicted by our model, wide-spread reduction of injection rates resulted in a wide-spread decrease of seismicity from 2016 to 2017 (red colour in **b**). Only in few areas, located at the edges of the earthquake zone, does the seismic activity slightly increase (blue colour in **b**).

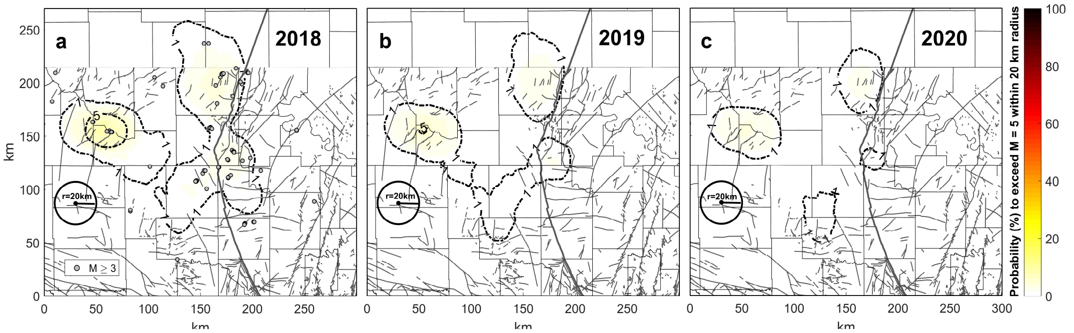

Supplementary Figure 11. Physics-based 1-year magnitude (M ≥ 5) exceedance probability forecasts (2018-2020). Exceedance probabilities are forecasted in areas of 1257 km^2 (20 km radius) and for the time of one year. The local seismic hazard is controlled by local pressure increase at depth and the local seismogenic state (the SI Fig. 2 b-e). 2018-2020 probabilities were computed assuming constant injection rates after March 2018. The maps can be produced for arbitrary future injection scenarios to optimize the distribution of fluid injection volumes in space and time for seismic hazard mitigation.

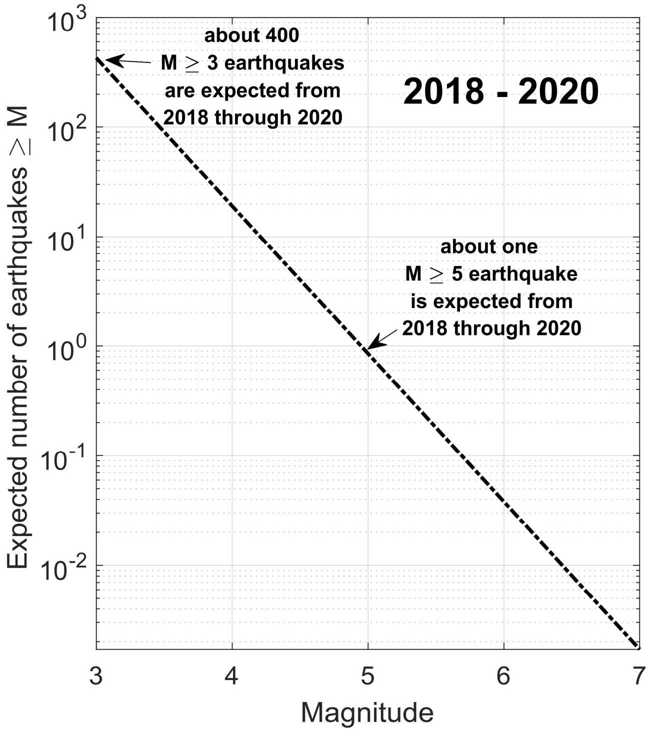

Supplementary Figure 12. Expected number of earthquakes of magnitude M or larger from Jan 2018 through Dec 2020. Expected numbers of earthquakes have been computed according to Eq.1 (see main text) and a constant injection level after March 2018. About 400 M ≥ 3 and one M ≥ 5 earthquakes are expected to occur from Jan 2018 through Dec 2020.

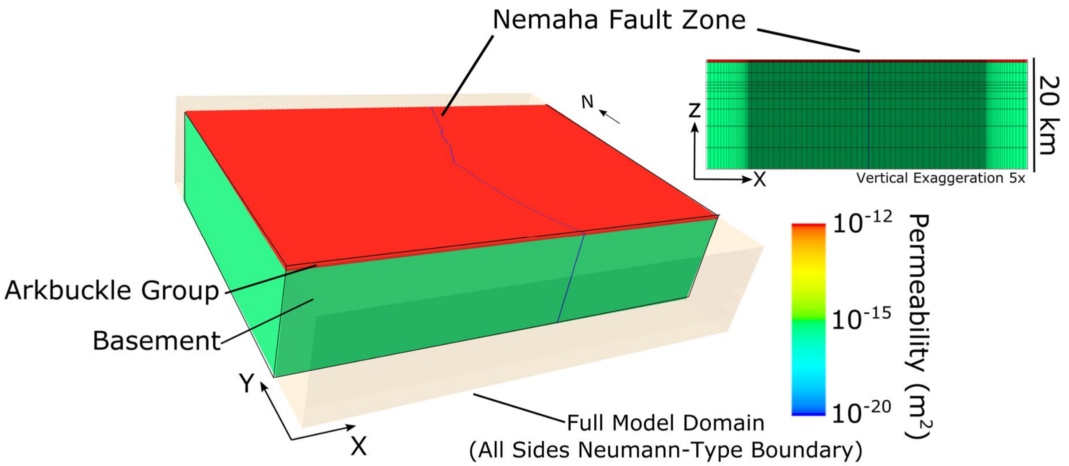

Supplementary Figure 13. Preferred hydrogeologic model domain, setup, grid discretization and boundary conditions. The hydrogeologic model consists of layered permeability heterogeneity with permeable Arbuckle Group sediments stratigraphically on top of lower permeability crystalline basement rock. The Nemaha Fault Zone is discretized as a zone of ultra-low permeability (i.e. regional flow barrier) in all model sensitivity analysis. All outer boundaries of the model domain are of Neumann-Type (no-flow) and set sufficiently far from in x and y from injection well locations to have no effect on model results. The horizontal discretization in x and y is 500 meters and consists of 12 discretization layers in the vertical direction.

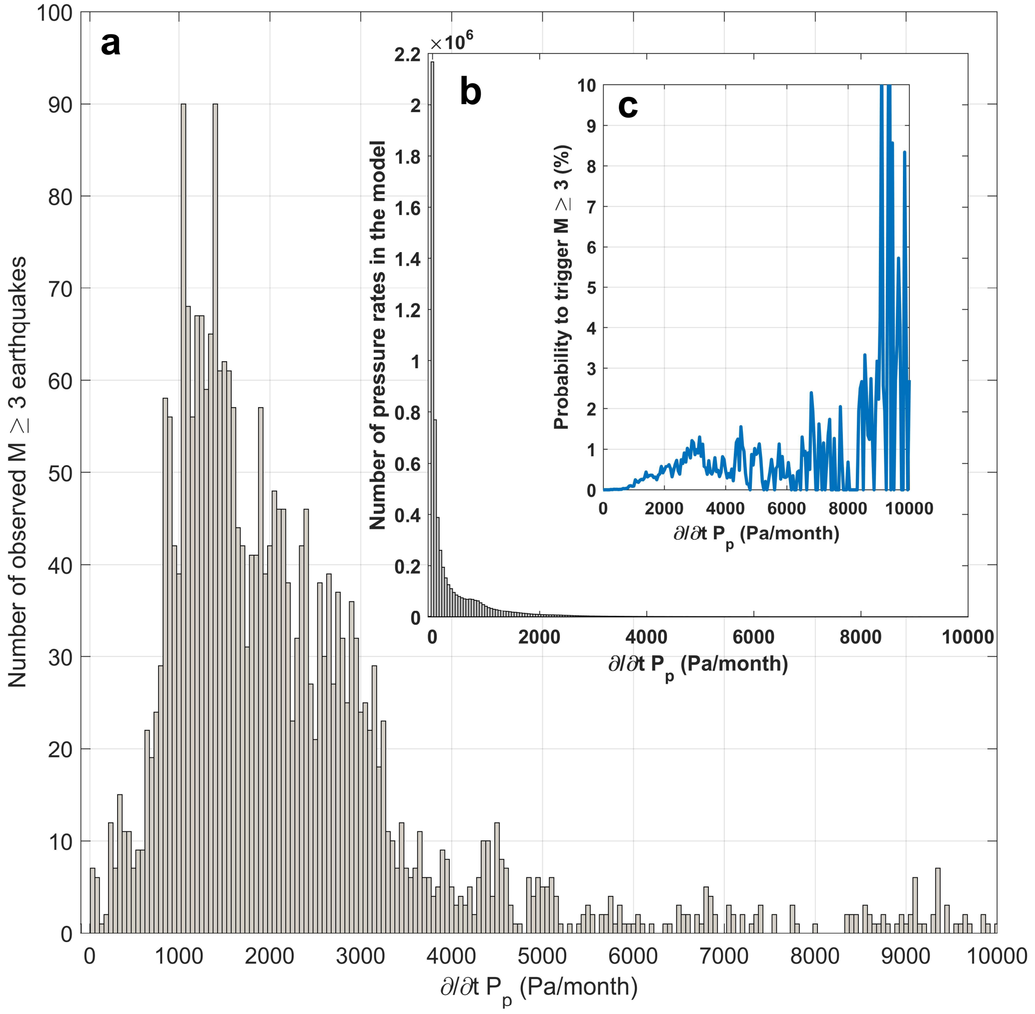

Supplementary Figure 14. Reconstructed probability to trigger M ≥ 3 earthquakes at a given pressure rate in our model. a, Monthly modelled pressure rates at occurrence times and hypocentres of observed M ≥ 3 earthquakes. **b**, Histogram of all monthly pressure rates in the model (at the 25,000 fault seed points). **c**, Reconstructed probability to trigger a fault seed point (M ≥ 3) at a given pressure rate in the model. The probability in **c** was computed by dividing the histogram shown in **a** by the histogram shown in **b**. Pressure rates larger than 3200 Pa/month represent less than 1% in the model. These rates occur only very locally in the Anadarko shelf region, which is characterized by a low SI (see Supplementary Fig. 6). It explains the lower triggering probabilities for higher pressure rates.

Supplementary References:

1. Carr, J. E., McGovern, H. E., Gogel, T. & Doveton, J. H. Geohydrology of and potential for fluid disposal in the Arbuckle aquifer in Kansas, Open File Report 86-491, US Geological Survey, (1986).

2. Perilla, P. C. Rock properties derived from analysis of earth tide strain observed in continuous pressure monitoring of the Arbuckle Group of Oklahoma, University of Oklahoma, M. S. Thesis, 79 p., (2017).

3. Freeze, R. A. & Cherry, J. A. Groundwater. Prentice-Hall, Englewood, Cliffs, New Jersey, 604 pp., (1979).
4. Nelson, P. H., Gianoutsos, N. J. & Drake., R. M. Underpressure in Mesozoic and Paleozoic rock units in the Midcontinent of the United States Underpressure in Mesozoic and Paleozoic Rock Units in the Midcontinent of the United States. AAPG Bulletin **99 (**10), 1861-1892, (2015).
5. Kroll, K.A., Cochran, E.S. & Murray, K.E. Poroelastic Properties of the Arbuckle Group in Oklahoma Derived from Well Fluid Level Response to the 3 September 2016 Mw 5.8 Pawnee and 7 November 2016 Mw 5.0 Cushing Earthquakes. Seismological Research Letters, **88** (4): 963-97, (2017).
